# Diagnostic performance of breast tumor tissue selection in diffusion weighted imaging: A systematic review and meta-analysis

**DOI:** 10.1371/journal.pone.0232856

**Published:** 2020-05-06

**Authors:** M. Wielema, M. D. Dorrius, R. M. Pijnappel, G. H. De Bock, P. A. T. Baltzer, M. Oudkerk, P. E. Sijens

**Affiliations:** 1 Department of Radiology, University of Groningen, University Medical Center Groningen, Groningen, The Netherlands; 2 Department of Radiology, Utrecht University, University Medical Center Utrecht, Utrecht, The Netherlands; 3 Department of Epidemiology, University of Groningen, University Medical Center Groningen, Groningen, The Netherlands; 4 Department of Biomedical Imaging and Image-guided Therapy, Medical University of Vienna, Vienna, Austria; 5 University of Groningen, Groningen, The Netherlands; 6 Institute for Diagnostic Accuracy, Groningen, The Netherlands; Henry Ford Health System, UNITED STATES

## Abstract

**Background:**

Several methods for tumor delineation are used in literature on breast diffusion weighted imaging (DWI) to measure the apparent diffusion coefficient (ADC). However, in the process of reaching consensus on breast DWI scanning protocol, image analysis and interpretation, still no standardized optimal breast tumor tissue selection (BTTS) method exists. Therefore, the purpose of this study is to assess the impact of BTTS methods on ADC in the discrimination of benign from malignant breast lesions in DWI in terms of sensitivity, specificity and area under the curve (AUC).

**Methods and findings:**

In this systematic review and meta-analysis, adhering to the PRISMA statement, 61 studies, with 65 study subsets, in females with benign or malignant primary breast lesions (6291 lesions) were assessed. Studies on DWI, quantified by ADC, scanned on 1.5 and 3.0 Tesla and using b-values 0/50 and ≥ 800 s/mm^2^ were included. PubMed and EMBASE were searched for studies up to 23-10-2019 (n = 2897). Data were pooled based on four BTTS methods (by definition of measured region of interest, ROI): BTTS1: whole breast tumor tissue selection, BTTS2: subtracted whole breast tumor tissue selection, BTTS3: circular breast tumor tissue selection and BTTS4: lowest diffusion breast tumor tissue selection. BTTS methods 2 and 3 excluded necrotic, cystic and hemorrhagic areas. Pooled sensitivity, specificity and AUC of the BTTS methods were calculated. Heterogeneity was explored using the inconsistency index (I^2^) and considering covariables: field strength, lowest b-value, image of BTTS selection, pre-or post-contrast DWI, slice thickness and ADC threshold. Pooled sensitivity, specificity and AUC were: 0.82 (0.72–0.89), 0.79 (0.65–0.89), 0.88 (0.85–0.90) for BTTS1; 0.91 (0.89–0.93), 0.84 (0.80–0.87), 0.94 (0.91–0.96) for BTTS2; 0.89 (0.86–0.92), 0.90 (0.85–0.93), 0.95 (0.93–0.96) for BTTS3 and 0.90 (0.86–0.93), 0.84 (0.81–0.87), 0.86 (0.82–0.88) for BTTS4, respectively. Significant heterogeneity was found between studies (I^2^ = 95).

**Conclusions:**

None of the breast tissue selection (BTTS) methodologies outperformed in differentiating benign from malignant breast lesions. The high heterogeneity of ADC data acquisition demands further standardization, such as DWI acquisition parameters and tumor tissue selection to substantially increase the reliability of DWI of the breast.

## Introduction

Breast magnetic resonance imaging (MRI) is mainly used as a problem solving, screening (in high risk patients) and monitoring tool and in pre-operative staging. In breast cancer diagnosis, dynamic contrast enhanced (DCE) MRI, while known for its high sensitivity near 100%, has a variable positive predictive value leading to a substantial proportion of unnecessary biopsies [1,2]. DCE-MRI alone regularly cannot provide certainty whether an enhancing lesion is benign. Diffusion Weighted Imaging (DWI) is now widely used as an important addition to standard breast MRI protocol to improve specificity and avoid unnecessary biopsies in benign enhancing breast lesions [3,4].

Optimal use of DWI requires quantification, which can be performed with different models. In the clinic, the mono-exponential model, yielding a single apparent diffusion coefficient (ADC) as a measure of water diffusion within the examined voxels, is most wide spread, due to its simplicity and availability. Standardization of acquisition parameters and breast tumor tissue selection (BTTS) methods (by definition of a region of interest (ROI)) is needed to measure ADC reliably and accurately. Acquisition protocol standardization is an ongoing topic with global attention, for example the recent recommendations of an international DWI working group supported by EUSOBI are presented in a consensus statement on breast DWI [5]. Several factors that could influence the ADC have already been evaluated; the choice of b-values influences the ADC, with highest accuracy for b-values 0–800 and 0–1000 s/mm^2^ [6]. No influence of pre-administered contrast agent on ADC was found [6]. Additionally, diagnostic accuracy of DWI was not significantly different between 1.5 or 3.0 T scanners [7]. Other scanning parameters, such as fat saturation, signal to noise ratio and partial volume effects (slice thickness and spatial resolution) also influence the accuracy of ADC [8,9]. Although, the diagnostic performance of DWI data processing using different BTTS methods has been addressed, to date little uniformity is seen in breast tumor tissue selection in the literature and no evidence-based recommendation on this specific topic is available yet. In recent clinical studies, BTTS is generally performed on post-contrast T1-weighted images or DWI-images/ADC maps. BTTS methods vary between areas covering the whole lesion to small sub-regions of low ADC, with methods based on selected areas in between [10]. A recent meta-analysis on breast DWI barely mentioned BTTS methods and did not provide thorough definitions and accuracy analysis of BTTS methods [11].

Therefore, this systematic review and meta-analysis primarily assesses the impact of different defined breast tumor tissue selection methods on the accuracy of ADC in terms of sensitivity, specificity and area under the curve (AUC) to distinguish benign from malignant breast lesions, with the intention to optimize breast DWI for routine application in clinical practice.

## Materials and methods

### Protocol and eligibility criteria

This study has been performed using the PRISMA statement (S1 Checklist) [12], applying Domain: (human) breast lesions visible on MRI; Determinant: Breast tumor tissue selection methods in DWI and Outcome: sensitivity, specificity and area under the summarized ROC curve (AUC). On 23-10-2019 a search was performed in Medline and Embase (S1 File).

### Search strategy

(“Breast Neoplasms”[Mesh] OR "Breast"[Mesh] OR mamma carcin*[tiab] OR mammary[tiab] OR mammo*[tiab] OR breast[tiab]) AND (“Diffusion Magnetic Resonance Imaging”[Mesh] OR "Diffusion Tensor Imaging"[Mesh] OR DWI[tiab] OR diffusion-weighted imag*[tiab] OR (("Diffusion"[Mesh] OR diffusion[tiab]) AND (“Magnetic Resonance Imaging”[Mesh] OR MRI[tiab] OR magnetic resonan*[tiab] OR MR[tiab] OR NMR[tiab]))).

### Study selection

Duplicates and studies before the year 2000 were excluded using Mendeley (Desktop 1.17.11). Study inclusion and data collection was performed by two readers independently (MW and MDD). Disagreements were resolved by a third reader (PES). Inclusion criteria were: 1) English language. 2) Breast DWI. 3) Human subjects. 4) Inclusion of both benign and malignant primary lesions. 5) Pathology or follow-up of benign lesions of at least 1 year was used as a reference standard. 6) 1.5 or 3.0 Tesla. 7) At least 2 b-values (lowest 0 or 50 s/mm^2^ and highest ≥ 800 s/mm^2^. 8) ADC threshold was mentioned. 9) TP, FN, FP and TN could be directly extracted or could be calculated from group numbers and diagnostic estimates such as sensitivity and specificity. 10) ADC threshold was chosen to acquire highest sensitivity and specificity combination. 11) Clearly described BTTS methods. 12) No high risk of bias in QUADAS-2 item assessment. Only mean lesion ADC values were included of original studies. When the same data was used in different papers, the paper with the highest patient number was included. Three studies with ADC thresholds biased by maximum sensitivity selection were excluded to avoid bias by threshold effect [4,9,13]. This approach differs from the Youden-index based best sensitivity/specificity pair’s method, as is used in the majority of studies. Studies with non-mass lesions only were excluded [14].

### Data collection

The following data were extracted: author, year of publication, number of lesions (benign/malignant), field strength, slice thickness, pre-or post -contrast DWI, image of BTTS drawing, b-values, ADC threshold, sensitivity, specificity and number of false positive (FP), false negative (FN), true positive (TP) and true negative (TN) outcomes. For data of two readers presented in a paper, mean values of both readers were included [15–18]. From multiple b-value combinations [19–23] or multiple cut-off values [3,24,25] presented, the outcome showing highest combination of sensitivity and specificity was included. When both mass and non-mass lesions were analyzed separately, only mass lesions were included [3,16,26,27]. In case two different DWI sequences were compared, the sequence most comparable to other papers was included (spectral fat suppression instead of STIR [8] and only EPI DWI [28]), which is in accordance with international recommendations [5].

### BTTS method categorization

Breast tumor tissue selection methods of included study subsets were categorized based on the following descriptions: BTTS1) Whole breast tumor tissue selection, as large as possible or slightly smaller than lesion size, excluding normal breast tissue, generally freeform. BTTS2) Subtracted whole breast tumor tissue selection, whereby the BBTS covered the whole lesion, without regions of necrosis, cystic and hemorrhagic areas. This included BTTSs covering the whole hyper/hypo-intense area on DWI/ADC-map, whereby areas of less diffusion restriction, which are likely to represent necrosis, hemorrhage, cystic areas or normal fibroglandular tissue were not included. BTTS3) Circular breast tumor tissue selection, i.e. one or multiple round or elliptical BTTSs, while avoiding necrosis and cystic or hemorrhagic areas. BTTS4) Lowest diffusion breast tumor tissue selection: Specifically selected area of lowest diffusion or brightest/darkest part of the lesion on the respective DWI/ADC map or the solid/most enhancing portion on DCE images. All study subsets were categorized by MW, MDD and PES independently and discussed until consensus was reached.

### Statistical analysis

A contingency table was made to asses FP, FN, TP and TN values of each study subset. Sensitivity and specificity were plotted in forest plots, including pooled sensitivity and specificity per BTTS category. Summarized Receiver Operating Characteristic (SROC) curves were made for each of the 4 BTTS categories and the AUC was calculated. The inconsistency index: “I^2^ = 100% x (Q—df)/Q” (Q = Cochran's heterogeneity statistic, df = degrees of freedom) was used to test for heterogeneity. I^2^ is a measure of heterogeneity, independent from the number of included studies, describing the percentage of inter-study variation due to heterogeneity. An I^2^ value of greater than 50% was considered as substantial heterogeneity [29]. Risk of publication bias was tested using Deeks’ Funnel Plot (linear regression of log odds ratios on inverse root of effective sample sizes as a test for funnel plot asymmetry). Meta regression analysis was performed on the following co-variables: 1) field strength (1.5T vs. 3.0T), 2) lowest b-value (0 vs. 50 s/mm2), 3) image of BTTS drawing: sequence on which the BTTS was drawn (DWI/ADC vs. DCE (or with reference to DCE)), 4) pre- or post-contrast DWI, 5) slice thickness (continuous, mm) and 6) ADC threshold (continuous, mm^2^/s). Meta-regression analysis was performed with a random effect model, to estimate the extent to which the co-variables are a potential source of heterogeneity in the data. Additional pooled sensitivity, specificity and AUC were calculated for each 1.5T and 3.0T separately. Midas, Metan and Metareg packages in STATA SE 14 were used. A p-value of <0.05 was considered to show a significant difference.

### Quality assessment

QUADAS-2 (Quality Assessment of Diagnostic Accuracy Studies) analysis was performed by two reviewers (MW and MDD) independently [30]. Consensus was obtained by discussion between (MW) and (MDD). Results of the QUADAS-2 analysis were visualized in Review Manager (RevMan 5.3.Ink, Cochrane Community).

## Results

### Study selection

**Fig 1:**
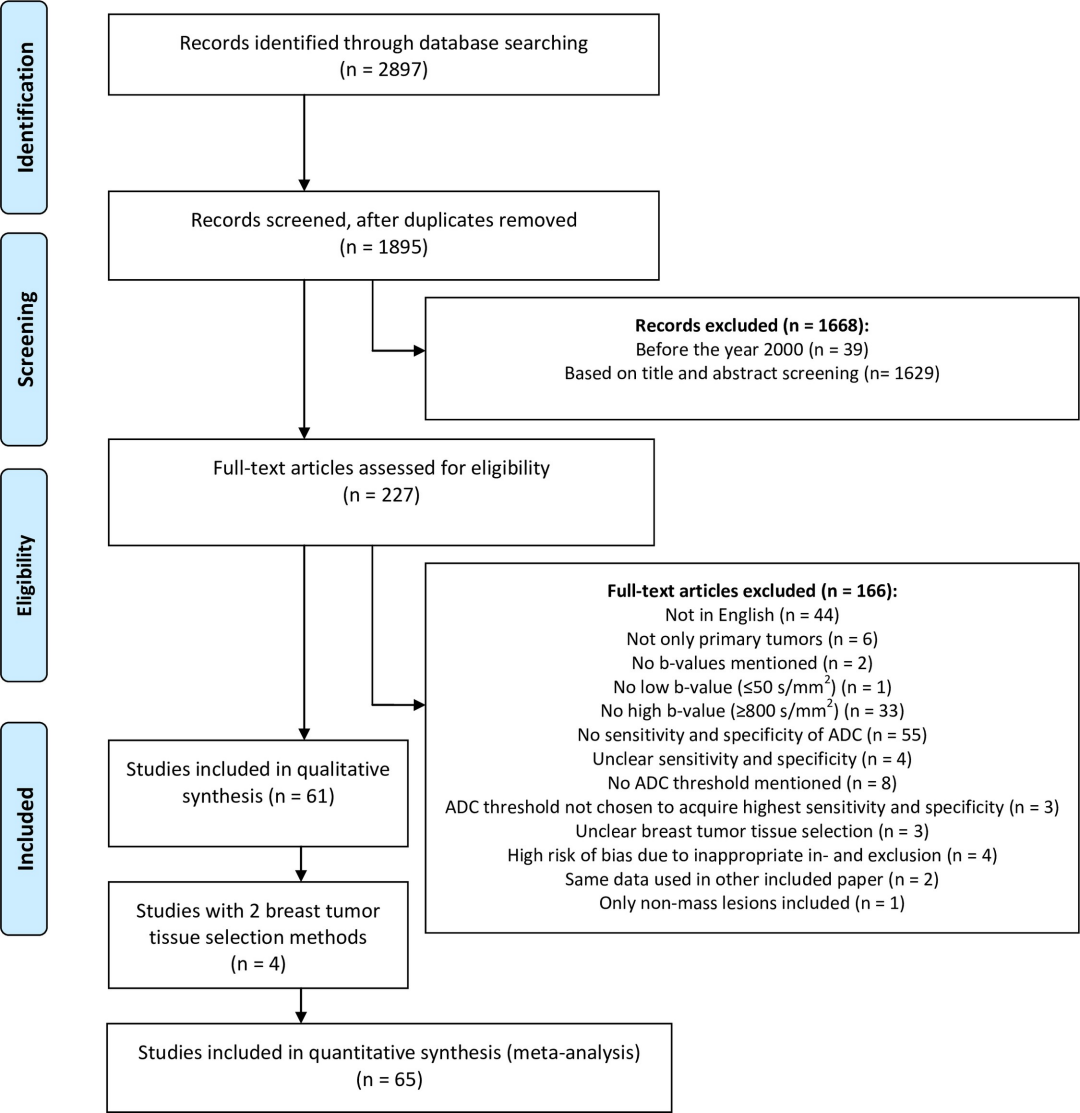
Flowchart of the study selection process, reasons for excluding papers and numbers of excluded and included papers. ADC = apparent diffusion coefficient, BTTS = breast tumor tissue selection.

By applying the aforementioned search in PubMed and Embase, 2897 papers were found of which 39 were published before the year 2000. Duplicates (n = 1002) were removed. Of the remaining 1895 articles, 1668 were excluded based on carefully applying the exclusion criteria on titles and abstracts. Of the remaining 227 fully read articles, four papers were excluded based on high risk of bias in one of the categories in the QUADAS-2 analysis, due to inappropriate exclusions implying selection bias [31–34]. Sixty-one eligible articles were identified to be analyzed of which four articles featured two breast tumor tissue selection methods, which were both included [16,35–37]. Thereby, a total of 65 study subsets were included in this systematic review and meta-analysis. The flowchart (Fig 1) shows the exclusion of articles based on the pre-defined exclusion criteria.

### Characteristics of the studies included

A total of 6291 lesions were found in the 65 included study subsets, of which 3982 were malignant and 2309 benign. The number of malignant and benign lesions ranged from 14–259 and 8–161, respectively, with a mean of 61 malignant and 36 benign lesions per study subset. Seven study subsets were categorized as BTTS1: whole breast tumor tissue selection [35,38–43], 22 as BTTS2: subtracted whole breast tumor tissue selection [15–17,20,23,36,37,44–58], 19 as BTTS3: circular breast tumor tissue selection [18,19,21,24,25,36,59–71] and 17 as BTTS4: lowest diffusion breast tumor tissue selection [3,8,16,22,26–28,35,37,72–79]. Table 1 provides an overview of the numbers of lesions (benign/malignant), field strength, slice thickness, DWI pre/post contrast, image of BTTS drawing, b-values (s/mm^2^) used for ADC calculation and ADC thresholds (10^−3^ mm^2^/s), for all study subsets included, categorized per BTTS method (alphabetical order). Thirty-nine study subsets used 1.5T and 26 study subsets 3.0T MRI. Slice thickness varied from 2.5 to 8 mm. Diagnostic ADC thresholds varied from 0.690–1.600 mm^2^/s, with a mean of 1.164 mm^2^/s (Table 2).

**Table 1:** Characteristics of the included study subsets grouped per breast tumor tissue selection (BTTS) method.

	**BTTS 1:** **Whole tumor tissue selection:**											
**Author + year**	**Reference**	**Number of lesions**	**Number of malignant lesions**	**Number of benign lesions**	**Field strength (Tesla)**	**Slice thickness (mm)**	**DWI pre/post contrast**	**Image of BTTS drawing**	**b-values used for ADC calculation (s/mm**^**2**^**)**	**ADC threshold (10**^**−3**^ **mm**^**2**^**/s)**	**Sensitivity**	**Specificity**
Bougias 2017	[42]	53	34	19	1.5	4	Pre	DWI, copied to ADCmap	0, 1300	1.150	0.97	0.95
Hirano 2012 (1)	[35]	75	48	27	3.0	3	Pre	ADCmap	50, 800, 1500	0.980	0.85	0.85
Jin 2010	[39]	60	40	20	1.5	4	Pre	DWI	0, 1000	1.180	0.77	0.95
Li 2019	[43]	120	62	58	3.0	5	Pre	DWI	0, 1000	1.091	0.63	0.90
Satake 2011	[40]	115	88	27	3.0	3	Pre	ADCmap, with reference to DCE	50, 1500	0.910	0.69	0.70
Song 2017	[41]	131	106	25	3.0	3	Pre	ADCmap	0, 1000	1.110	0.87	0.68
Woodhams 2005	[38]	191	167	24	1.5	6	Pre	ADCmap, with reference to DCE	0, 750, 1000	1.600	0.93	0.46
	**BTTS 2:** **Subtracted whole breast tumor tissue selection:**											
Aribal 2016	[49]	138	75	63	1.5	3	Pre	ADCmap, with reference to DCE	50, 800	1.118	0.91	0.83
Arponen 2015 (1)	[16]	137	114	23	3.0	4	Post	ADCmap	0, 200, 400, 600, 800	0.870	0.93	0.78
Arponen 2016	[47]	29	14	15	3.0	4	Post	ADCmap	0, 200, 400, 600, 800	0.870	0.93	0.80
Bogner 2009	[20]	61	24	17	3.0	3.5	Pre	DWI, with reference to DCE + T2	50, 850	1.250	0.96	0.94
Bogner 2012	[17]	49	28	21	3.0	5	Pre	ADCmap, with reference to DWI, DCE	0, 850	1.250	0.89	0.90
Chen 2018	[55]	116	72	44	3.0	5.5	Post	DCE, copied to ADCmap	50, 800	1.240	0.76	0.62
Fanariotis 2018	[23]	62	27	35	3.0	4	Pre and Post	DWI, with reference to DCE	0, 1000	1.100	0.93	0.89
Gity 2018 (1)	[37]	98	50	48	1.5	5	Post	ADCmap	0, 800	1.450	0.92	0.74
Gruber 2016	[46]	28	18	10	3.0	5	NA	DWI, with reference to ADCmap	0, 850	1.257	0.94	0.90
Guo 2002	[56]	47	30	17	1.5	5	Pre	DWI/ADC	0, 250, 500, 750, 1000 AND 0, 1000	1.300	0.93	0.88
Iima 2015	[53]	23	15	8	3.0	3	Pre	DWI, with reference to T2	0, 1000	1.400	1.00	0.88
Inoue 2011	[57]	106	91	15	1.5	8	Pre	ADCmap	0, 250, 500, 750, 1000	1.290	0.95	0.80
Liu 2013	[52]	111	40	41	1.5	5	Pre	ADCmap, with reference to MRI (incl. DCE)	0, 1000	1.180	0.93	0.90
Moschetta 2014	[44]	128	77	51	1.5	3	Pre	DWI	0, 1000	1.440	0.97	0.83
Pinker 2013	[58]	294	209	85	3.0	3.5	Pre	ADCmap, with reference to DWI, DCE, MRI	50, 850	1.275	0.92	0.89
Pinker 2014	[54]	76	53	23	3.0	3.5	Pre	ADCmap, with reference to DWI, DCE	50, 850	1.250	0.96	0.96
Rubesova 2006	[15]	87	65	22	1.5	4	Post	ADCmap, with reference to DCE	0, 200, 400, 600, 1000	1.150	0.85	0.86
Si 2016	[48]	57	28	29	3.0	4	Pre	ADCmap, with reference to DCE	0, 800	1.190	0.71	0.86
Sun 2015	[51]	98	57	41	1.5	4	Pre	ADCmap, with reference to DWI, DCE	50, 1000	0.895	0.86	0.83
Suo 2017	[50]	101	57	44	3.0	3	Pre	DWI, with reference to MRI	0, 10, 30, 50, 100, 150, 200, 500, 800, 1000, 1500, 2000, 2500	0.870	0.93	0.75
Wan 2016	[45]	95	74	21	1.5	NA	Pre	DCE, copied to ADCmap	0, 1000	1.088	0.86	0.78
Zhang 2015 (1)	[36]	248	163	85	1.5	6	Pre	ADCmap, with reference to DCE, T2, T1	0, 800	1.223	0.85	0.92
	**BTTS 3: C****ircular breast tumor tissue selection:**											
Abd El-Aleem 2018	[64]	44	25	19	1.5	3.5	Pre	ADCmap	0, 200, 400, 600, 800	1.260	0.88	0.95
Altay 2014	[62]	37	23	14	1.5	5	Pre	ADCmap, with reference to MRI	0, 1000	1.100	0.90	0.86
Cabuk 2015	[66]	63	22	41	1.5	4	Post	ADCmap	0, 1000	.980	0.86	0.81
Cakir 2013	[21]	55	30	25	3.0	2.5	Pre	ADCmap/DWI with reference to DCE	0, 1500	1.120	0.97	0.60
Fornasa 2011	[24]	78	35	43	1.5	7	NA	DWI	0, 800	1.480	0.89	0.95
Hatakenaka 2008	[68]	140	124	16	1.5	NA	Pre	ADCmap, with reference to MRI	0, 500, 1000	1.480	0.84	0.81
Ibrahim 2015	[59]	40	18	22	1.5	3	Post	ADCmap, with reference to MRI	0, 400, 800	1.250	1.00	0.77
Kothari 2017	[61]	88	65	23	3.0	4	Pre	DWI	0, 800	1.100	0.92	0.74
Lo 2009	[69]	31	20	11	3.0	6	Pre	ADCmap, with reference to DCE, DWI	0, 1000	1.210	0.90	0.91
Marini 2007	[25]	81	42	39	1.5	4	Pre	ADCmap	0, 1000	1.100	0.80	0.81
Ozgokce 2019	[67]	51	23	28	1.5	NA	NA	ADCmap	0, 200, 400, 600, 800, 1000	1.100	0.91	0.96
Pereira 2009	[19]	52	26	26	1.5	5	Post	ADCmap, with reference to MRI	0, 250, 500, 750, 1000	1.210	0.92	0.92
Sahin 2013	[71]	51	35	16	1.5	3.5	Pre	ADCmap, with reference to DCE + T2	50, 800	1.030	0.89	1.00
Sharma 2016	[60]	326	259	67	1.5	NA	Post	ADCmap	0, 500, 1000	1.123	0.93	0.91
Sonmez 2011	[70]	45	25	20	1.5	5	Pre	ADCmap, with reference to DCE	0, 50, 200, 500, 1000	1.000	0.96	1.00
Tan 2014	[65]	44	31	13	3.0	NA	Pre	DWI, with reference to DCE	0, 1000	1.220	0.91	0.92
Yili 2009	[18]	57	35	22	1.5	5	Pre	ADCmap	0, 1000	1.200	0.96	0.97
Zhang 2015 (2)	[36]	248	163	85	1.5	NA	Pre	ADCmap, with reference to DCE, T2, T1	0, 800	1.315	0.90	0.89
Zhao 2016	[63]	48	25	23	3.0	4	Pre	DWI, with reference to DCE	0, 1000	1.350	0.68	0.96
	**BTTS 4:** **Lowest diffusion breast tumor tissue selection:**											
**Author + year**	**Reference**	**Number of lesions**	**Number of malignant lesions**	**Number of benign lesions**	**Field strength (Tesla)**	**Slice thickness (mm)**	**DWI pre/post contrast**	**Image of BBTS drawing**	**b-values used for ADC calculation (s/mm**^**2**^**)**	**ADC threshold (10**^**−3**^ **mm**^**2**^**/s)**	**Sensitivity**	**Specificity**
Arponen 2015 (2)	[16]	137	114	23	3.0	NA	Post	ADCmap	0, 200, 400, 600, 800	0.690	0.96	0.70
Baltzer 2009	[28]	74	39	35	1.5	6	Post	DWI, copied to ADCmap	0, 750, 1000	1.233	0.87	0.83
Baltzer 2010	[79]	81	54	27	1.5	6	Post	ADCmap	0, 750, 1000	1.230	0.91	0.76
Cheng 2013	[26]	127	86	41	1.5	4	Pre	DWI, with reference to DCE	0, 1000	1.050	0.70	0.85
Gity 2018 (2)	[37]	98	50	48	1.5	5	Post	ADCmap	0, 800	1.450	0.92	0.74
Hirano 2012 (2)	[35]	75	48	27	3.0	NA	Pre	ADCmap	50, 800, 1500	0.840	0.75	0.74
Khattab 2018	[77]	26	15	11	1.5	5	Post	DWI	0, 1000	1.240	0.87	0.82
Kul 2011	[72]	84	47	37	1.5	3	Pre	ADCmap	50, 1000	0.920	0.91	0.86
Kul 2014	[27]	285	124	161	1.5	3	Pre	DWI, with reference to DCE	50, 400, 1000	0.900	0.95	0.80
Kul 2018	[74]	203	143	70	1.5	3 or 4	Pre	ADCmap	50, 400, 1000 or 0, 500, 1000	0.960	0.91	0.90
Liu 2018	[75]	150	77	73	1.5	4	Pre	ADCmap	0, 800	1.080	0.83	0.80
Nogueira 2014	[22]	157	89	68	3.0	5	Pre	DWI, with reference to DCE	50, 200, 400, 600, 800, 1000	1.470	0.78	0.85
Ohlmeyer 2019	[78]	72	46	26	3.0	2.5	Post	DWI, with reference to DCE	50, 800	1.230	1.00	0.92
Ouyang 2014	[8]	39	23	16	3.0	4	Pre	ADCmap, with reference to T2 (necrosis)	0, 800	1.230	0.87	0.88
Polat 2013	[73]	61	26	35	1.5	5	Pre	ADCmap, with reference to DCE + T2	50, 400, 800	1.220	0.96	0.89
Spick 2014	[3]	61	15	46	1.5	NA	Pre	ADCmap	50, 400, 800	1.580	0.95	0.82
Yilmaz 2018	[76]	88	34	54	1.5	4.5	Post	DCE, copied to ADCmap	0, 1000	1.040	0.88	0.87

NA = not applicable, ADC = Apparent diffusion coefficient, DWI = diffusion weighted imaging, DCE = dynamic contrast enhanced, BTTS = breast tumor tissue selection and (1)/(2) refers to two different BTTS method inclusions (study subsets) from the same study.

**Table 2:** Pooled data categorized per BTTS method, including heterogeneity analysis.

BTTS method	Number of study subsets	Pooled sensitivity (95% CI)	Pooled specificity (95% CI)	AUC (95% CI)	Inconsistency index (I^2^)	Number of lesions	Number of malignant lesions	Number of benign lesions	ADC threshold (10^−3^ mm^2^/s) Min-Max (Range)	ADC threshold (10^−3^ mm^2^/s) Mean (sd)
**BTTS 1:**	7	0.82 [0.72–0.89]	0.79 [0.65–0.89]	0.88 [0.85–0.90]	93	745	545	200	0.910–1.600 (0.690)	1.155 (0.22)
**BTTS 2:**	22	0.91 [0.89–0.93]	0.84 [0.80–0.87]	0.94 [0.91–0.96]	0	2139	1381	758	0.870–1.450 (0.580)	1.179 (0.17)
**BTTS 3:**	19	0.89 [0.86–0.92]	0.90 [0.85–0.93]	0.95 [0.93–0.96]	77	1579	1026	553	0.980–1.480 (0.500)	1.191 (0.14)
**BTTS 4:**	17	0.90 [0.86–0.93]	0.84 [0.81–0.87]	0.86 [0.82–0.88]	86	1828	1030	798	0.690–1.580 (0.890)	1.122 (0.22)
**Total:**	65				95	6291	3982	2309	0.690–1.600 (0.910)	1.164 (0.18)

CI = confidence interval, ADC = apparent diffusion coefficient, BTTS = breast tumor tissue selection. BTTS1: whole breast tumor tissue selection, BTTS2: subtracted whole breast tumor tissue selection, BTTS3: circular breast tumor tissue selection and BTTS4: lowest diffusion breast tumor tissue selection.

### Diagnostic performance of BTTS methods

**Fig 2:**
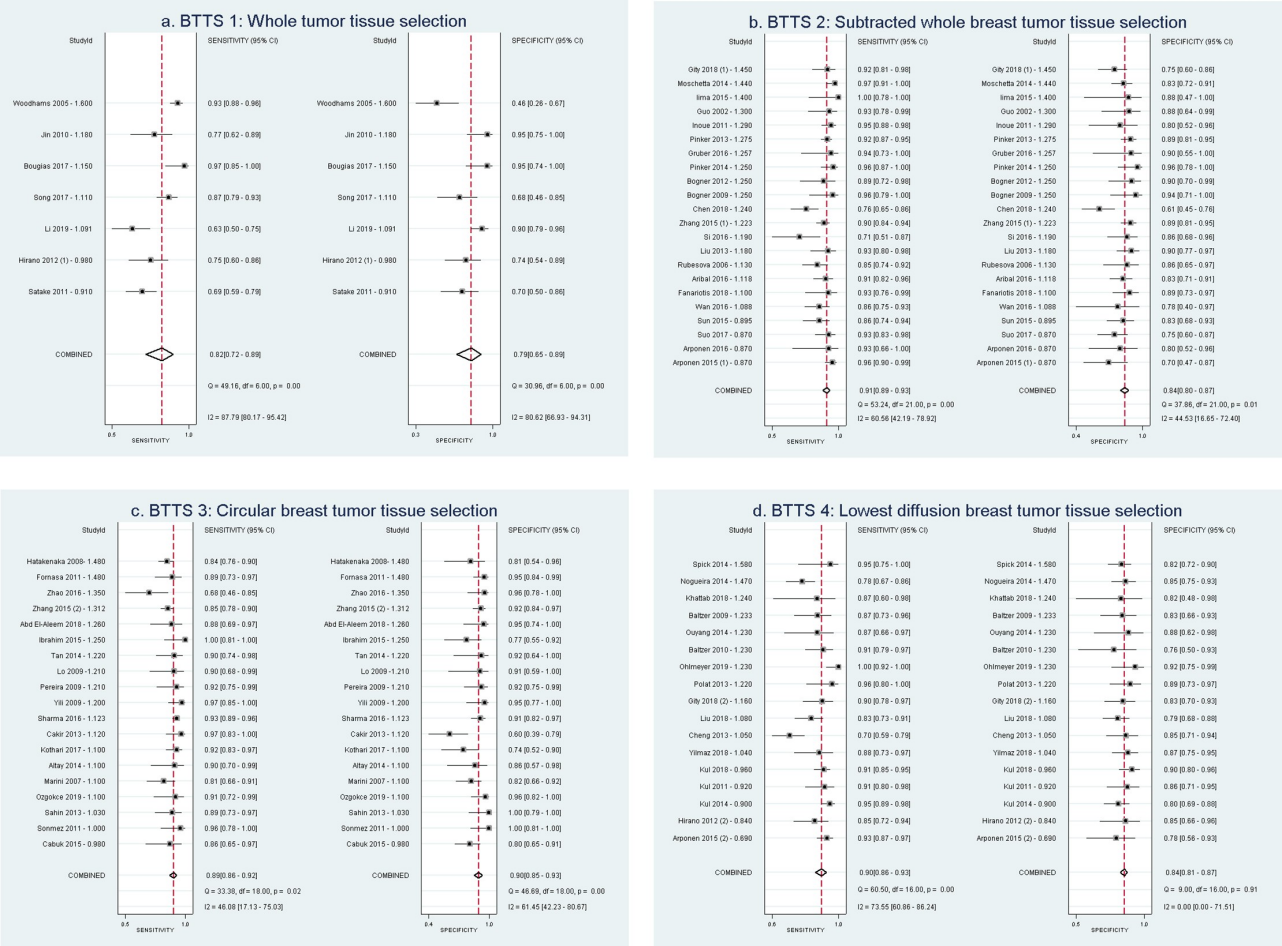
Forest plots for each BTTS method, showing sensitivity and specificity with confidence intervals of each study subset and in the last row the pooled values (diamond). Study subsets are sorted by ADC threshold. Study ID = Author—ADC threshold (10–3 mm2/s). a) BTTS1: Whole breast tumor tissue selection, b) BTTS2: Subtracted whole breast tumor tissue selection, c) BTTS3: Circular breast tumor tissue selection, d) BTTS4: Lowest diffusion breast tissue selection. BTTS = breast tumor tissue selection. (1)/(2) refers to the two different BTTS method inclusions (study subsets) from the same study.

**Fig 3:**
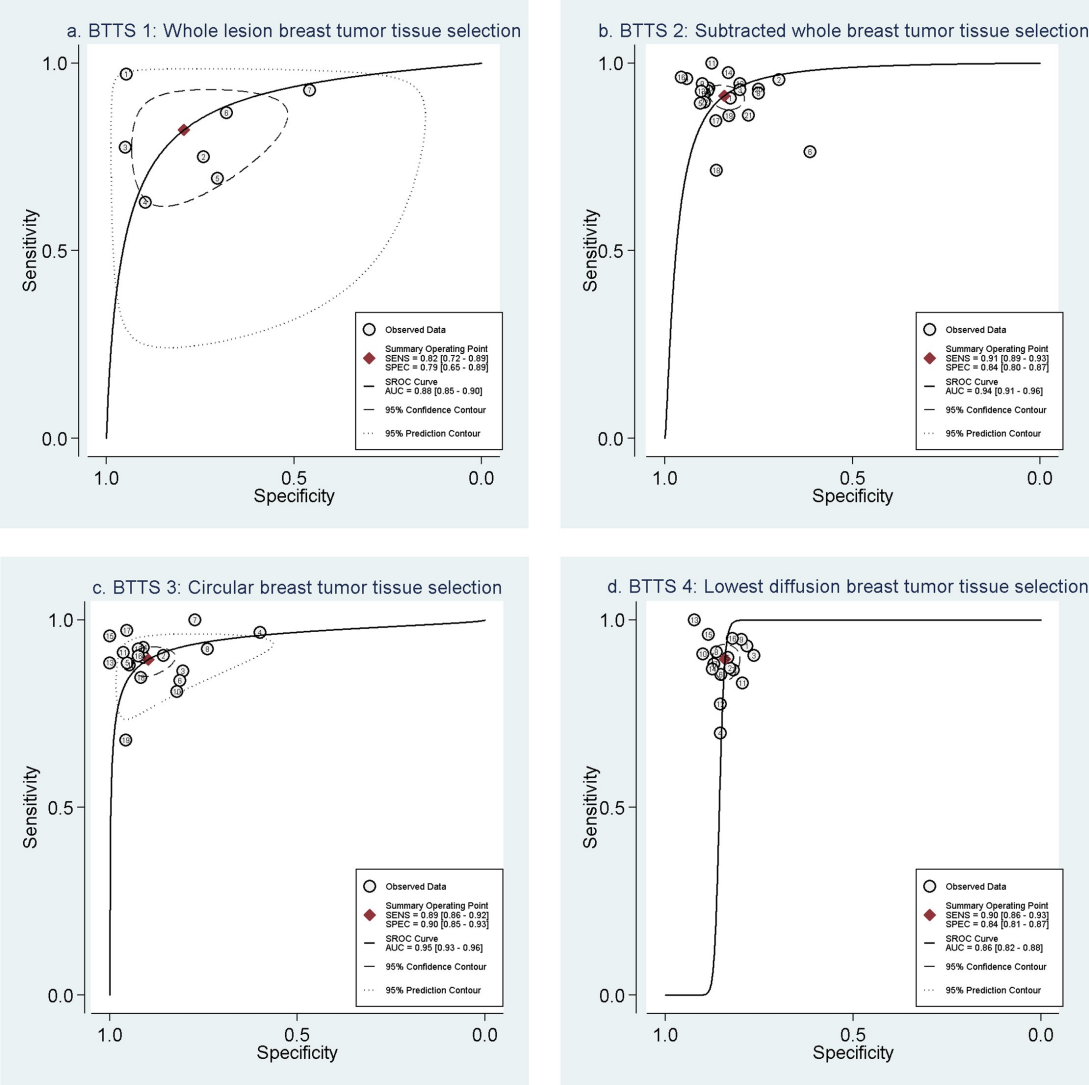
Summary Receiver Operating Curves of the diagnostic performance of each BTTS method. **SROC with prediction & confidence contours.** a) BTTS1: Whole breast tumor tissue selection, b) BTTS2: Subtracted whole breast tumor tissue selection, c) BTTS3: Circular breast tumor tissue selection, d) BTTS4: Lowest diffusion breast tissue selection. BTTS = breast tumor tissue selection, SROC = summarized receiver operator characteristic, AUC = area under the SROC curve, SENS = sensitivity, SPEC = specificity.

Table 1 and the forest plots (Fig 2A–2D) show the sensitivity and specificity of each study subset per BTTS method category. For each method, the forest plot is sorted based on ADC threshold (highest to lowest). Sensitivity and specificity of all inclusions ranged from 63–100% and 46–100%, respectively. Table 2 shows pooled sensitivity and specificity per BTTS method and AUC. Best combination of pooled sensitivity and specificity is found for BTTS3: circular breast tumor tissue selection, with values of 0.89 (Confidence Interval (CI): 0.86–0.92) and 0.90 (CI: 0.85–0.93). BTTS1 shows lowest pooled sensitivity and specificity of 0.82 (0.72–0.89) and 0.79 (0.65–0.89), respectively. Considerable overlap exists between the confidence intervals of pooled sensitivity and specificity of all BTTS methods, which results in no significant difference between the four BTTS methods. With regard to overall performance, Table 2 and SROC curves (Fig 3A–3D) demonstrate highest AUC of 0.95 (CI: 0.93–0.96) for BTTS3, compared to AUCs of 0.94 (CI: 0.91–0.96), 0.88 (CI: 0.85–0.90) and 0.86 (CI: 0.82–0.88) for BTTS2, BTTS1 and BTTS4, respectively. However, there is overlap of confidence intervals between BTTS2 and BTTS3.

### Risk of bias

**Fig 4:**
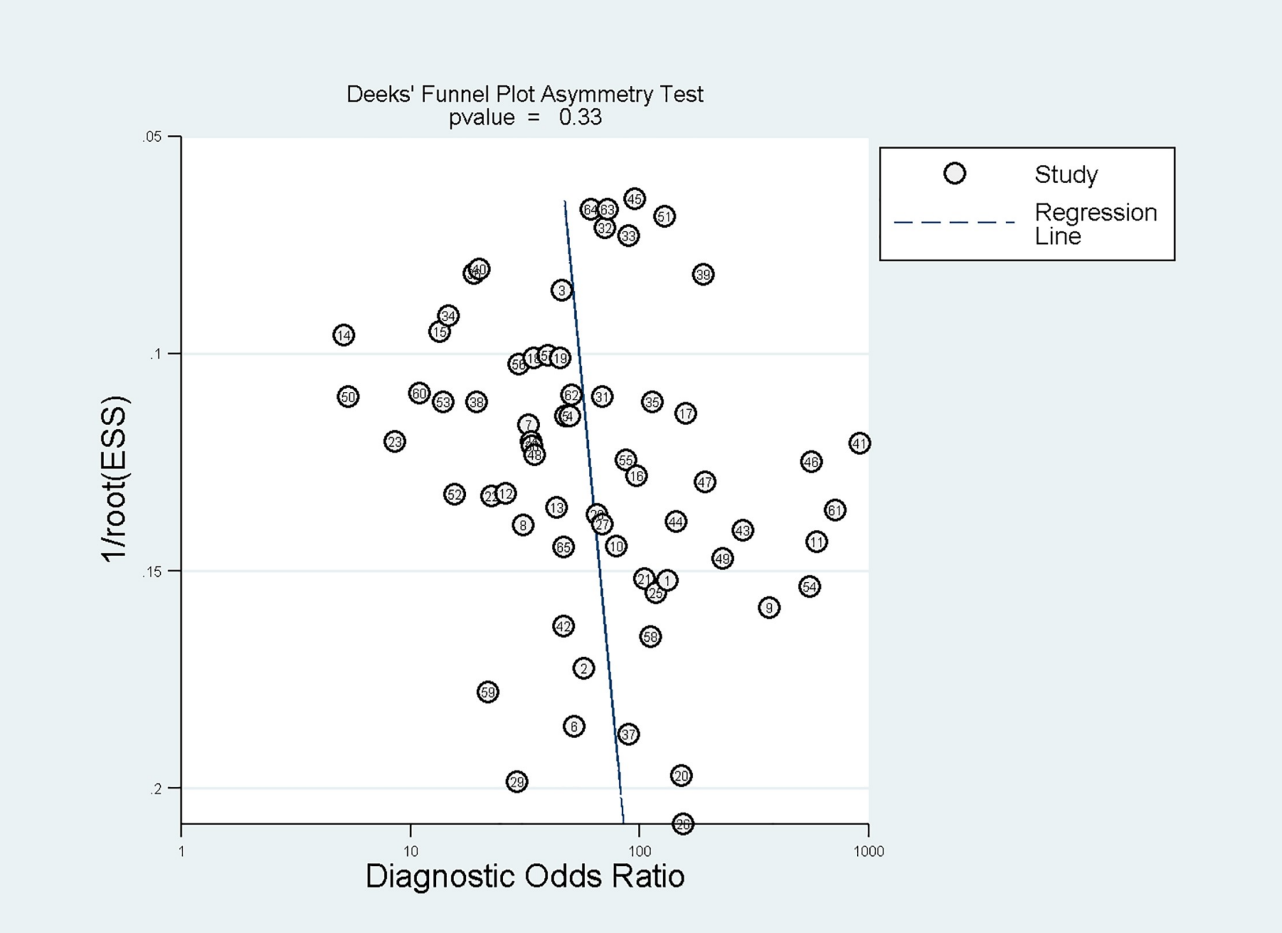
Deeks’ funnel plot shows no risk of publication bias.

An overview of measures of study subsets’ heterogeneity is given in Table 2. A significant heterogeneity (I^2^ = 95) was found when all included study subsets were considered together. Within the BTTS categories, no heterogeneity was present for BTTS2: subtracted whole breast tumor tissue selection (I^2^ of 0%). The other BTTS methods showed high heterogeneity (I^2^ = 93% (BTTS1), 77% (BTTS3) and 86% (BTTS4)). Deeks’ funnel plot with superimposed regression line, showed no risk of publication bias (p = 0.33) (Fig 4). The slope coefficient suggests symmetry in the data. Consequently, heterogeneity of all included papers is not likely to be due to publication bias.

### Meta-regression analysis

In the meta-regression analysis only field strength could explain part of the heterogeneity (p = 0.045, Table 3). Based on the meta-regression analysis results, a direct comparison of sensitivity and specificity of 1.5T vs. 3.0T was performed, which showed comparable sensitivity and specificity for 1.5 and 3.0T, with values of 0.90(0.88–0.92) and 0.86(0.84–0.88) for 1.5T and 0.89(0.85–0.92) and 0.83(0.79–0.87) for 3.0T, respectively (Table 4). None of the other co-variables influenced pooled sensitivity and specificity or could explain heterogeneity (Table 3). Data on co-variables were available in all papers for the field strength of the MRI scanner used, lowest b-value, image of BTTS drawing and ADC threshold used. Data on slice thickness were available for 55 of 65 included study subsets and for pre- or post-contrast DWI in 61 of 65 included study subsets. ADC thresholds were found comparable between the four BTTS categories, with the smallest range of 0.500 x 10^−3^ mm^2^/s for BTTS3 (Table 2).

**Table 3:** Meta-regression analysis of pooled data of all 65 study subsets using a random effect model.

Co-variable	Type of variable	Categories	p-value
Field strength (T)	Dichotomous	1.5 or 3.0 Tesla	0.045
Lowest b-value (s/mm^2^)	Dichotomous	0 or 50 s/mm^2^	0.288
Image of BTTS drawing	Dichotomous	Only ADC or DWI vs. ADC or DWI with reference to DCE	0.945
DWI pre or post contrast	Dichotomous	Pre- or post-contrast	0.326
Slice thickness (mm)	Continuous		0.762
ADC Threshold (mm^2^/s)	Continuous		0.429

ADC = apparent diffusion coefficient, DWI = diffusion weighted imaging, DCE = dynamic contrast enhanced, BTTS = breast tumor tissue selection and T = Tesla.

**Table 4:** Additional analysis: 1.5 vs. 3.0 Tesla, pooled sensitivity and specificity.

Field strength	Number of publications	Sensitivity (95% CI)	Specificity (95% CI)	AUC (95% CI)
1.5 Tesla	39	0.90 [0.88–0.92]	0.86 [0.84–0.88]	0.94 [0.92–0.96]
3.0 Tesla	26	0.89 [0.85–0.92]	0.83 [0.79–0.87]	0.92 [0.89–0.94]

CI = Confidence interval.

### Quality assessment using QUADAS-2 analysis

**Fig 5:**
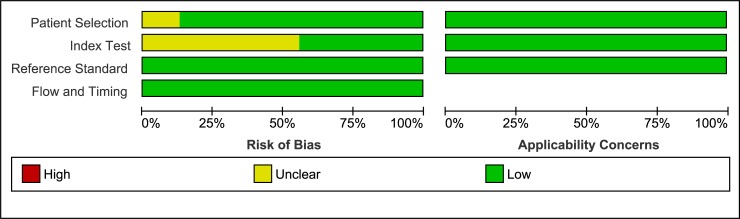
Summary of the risk of bias and applicability concerns on patient selection, index test, reference standard and flow and timing of the included papers, scored by the reviewers using QUADAS-2 analysis. QUADAS = Quality Assessment of Diagnostic Accuracy Studies.

**Fig 6:**
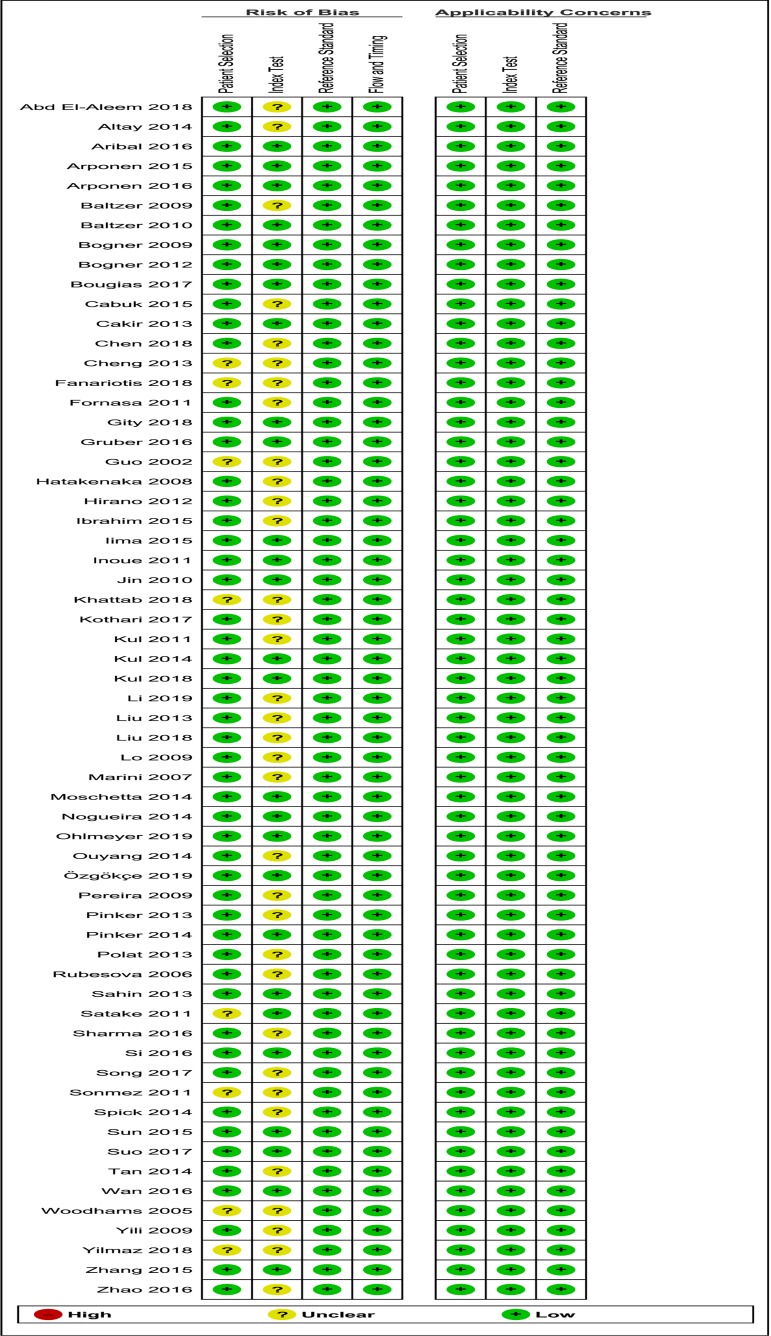
Overview of risk of bias and applicability concerns of each included paper, scored by the reviewers using QUADAS-2 analysis. QUADAS = Quality Assessment of Diagnostic Accuracy Studies.

Overall methodological quality of the included papers was moderate (Figs 5 and 6). The vast majority of papers did not mention whether the index test was interpreted without knowledge of the results of the reference standard. Moreover, the ADC threshold was not pre-specified in most papers, with the exception of 8 papers [17,20,44,46,47,54,67,79]. This resulted in a 56% bias risk in domain 2, regarding the Index test. Although patient selection was clear and without much concern of bias, three studies [38,56,76] were unclear about their in- and exclusion criteria and three other studies did not avoid inappropriate exclusions [26,40,70], for example by excluding bulky or superficial masses or by excluding patients in case of inconsistency between DCE and DWI. Three studies mentioned blinding of the pathologist [49,68,79]. Optimal score for “Reference standard” and “Flow and timing” was found for all studies included.

## Discussion

This is the first systematic review and meta-analysis of DWI studies focused on documenting the diagnostic accuracy of different breast tumor tissue selection methods to measure the ADC to distinguish between benign and malignant breast lesions. This meta-analysis shows the lack of uniformity in BTTS methods used for the calculation of ADC. The pooled data of the 65 included BTTS methods (of 61 included papers, addressing 6291 lesions), categorized into 4 BTTS methods, show considerable overlap between the confidence intervals of pooled sensitivity and specificity of all BTTS methods. However, the AUC of BTTS 2 and BTTS3 were higher than BTTS1 and BTTS4, without overlap of confidence intervals. Therefore, there seems to be a weak trend indicating that the selective methods excluding necrosis, hematomas and cysts could perform better than whole lesion or lowest diffusion BTTS methods. As evidenced by the objectivized levels of heterogeneity of ADC data acquisition, this study highlights the demand for further protocol standardization besides BTTS selection, such as DWI acquisition parameters to increase the reliability of DWI of the breast.

### Diagnostic performance

One may argue that a small selection of lowest ADC (BTTS4), which seems comparatively robust, objective and easiest to reproduce, would perform the best. However, our data does not support this hypothesis. The lack of outperformance of BTTS4 compared to the other BTTS methods reflects ultralow ADC values originating from non-suppressed fat, leading to a decrease in mean ADC [80]. In the other BTTS methods, where tissue selection is not based on the lowest ADC values, this effect will not prevail due to averaging. On the other hand, the more selective methods (BTTS2/BTTS3) apply visual selection by excluding necrosis, cystic areas and hemorrhage, based on the knowledge that these areas show little diffusion hindrance e.g. due to low cellularity and high water content [81]. Furthermore, partial volume effect on tumor edges in the more selective methods have been avoided, more than in methods with complete lesion coverage (BTTS1) or subregions (BTTS4). Therefore, the most straightforward method of searching for the lowest ADC fails to outperform.

### BTTS comparison

In the comparative literature on this topic, Zhang et al. showed a higher AUC of for BTTS (by definition of measured region of interest, ROI) covering the whole lesion, excluding necrosis etc. (BTTS2) compared to a spot measurement (BTTS3), 0.94 vs.0.92, respectively. However, these minor differences could not be confirmed by this meta-analysis (AUC:BTTS2:0.94; BTTS3:0.95, respectively) [36]. Arponen et al. showed AUCs for BTTS2 (0.96/0.85) and BTTS4 (0.96/0.89)(2 readers) comparable to the results of this meta-analysis [16]. Hirano et al. selected as many 25mm^2^ BTTSs as possible, of which the average of all BTTSs (BTTS1) showed a lower mean AUC (0.83) than their BTTS of lowest mean ADC (BTTS4: 0.93). These results are not supported by this meta-analysis (BTTS1:0.88, BTTS4:0.86) [35]. Some authors only studied the reproducibility of different BTTS methods. Bickel et al. studied different freehand BTTS methods. Their whole lesion and lowest diffusion BTTS showed higher AUC (0.94, 0.93, respectively), than the pooled estimates in this meta-analysis. The inter- and intra-reader agreement was equal for both methods. Sensitivity and specificity of this study were not provided [10]. Nogueira et al. concluded that a small BTTS in the area of highest DWI signal intensity showed higher reproducibility compared to whole lesion BTTS. No further test characteristics were provided [82].

### Study heterogeneity

The results of this meta-analysis apply to mass lesions only, because non-mass lesions were not included in most of the papers and the diagnostic accuracy of ADC in non-mass lesions is known to be limited [83]. Regarding the inclusion of studies, only one outcome per data set was included to avoid that overweighting would influence the outcome. For studies employing two different BTTS methods (n = 4), both were included in this meta-analysis [16,35–37]. Ouyang et al. compared conventional to STIR-DWI of which only conventional DWI data were included, being the most regularly applied method [8]. When multiple cut-off values were presented, the cut-off value with highest combination of both sensitivity and specificity was included [3,24,25].

When addressing between study heterogeneity, only 41 of 65 included study subsets provided a figure clarifying their BTTS method. Moreover, information on scanning parameters often was incomplete. Available parameters were assessed and most co-variables and did not influence pooled sensitivity and specificity. Pre-or post-contrast scanning did not show significantly different results in discriminating breast lesions, which is in accordance with literature [6]. Our results showed influence of field strength on pooled data. However, differences in sensitivity and specificity of 1.5 and 3.0T could not be proven due to the considerable overlap in confidence intervals, comparable to the study of Shi et al [7].

Furthermore, in most papers the ADC threshold was not pre-specified, which most probably can be explained by the heterogeneity in scanning parameters of the research sites. In a very recent general meta-analysis on DWI of breast lesions, including intravoxel incoherent motion (IVIM) and diffusion tensor imaging (DTI), BTTS methods were barely mentioned [11]. Moreover, broader inclusion criteria were used covering all b-value combinations. The breast tissue selection methodology was ill defined and poorly described, as whole lesion, small region of interest and single selection, without further description on for example the in/exclusion of necrotic tissue.

Another important covariable is image quality in terms of noise and fat suppression. Those covariables were scarcely reported in the included studies and therefore no conclusions can be drawn. Before any clinical implementation or adding DWI methodologies to the ACR BI-RADS lexicon, standardization of acquisition protocol as is addressed by the EUSOBI DWI working group [5] as well as image analysis, such as breast tumor tissue selection methods, is of high importance. This meta-analysis demonstrates the problematic issues to come to any standardization of DWI BTTS methods. Our data shows no superiority of any BTTS method, therefore an evidenced based conclusion cannot be drawn. Furthermore, this meta-analysis shows that complete and accurate reporting of acquisition parameters is obligatory for any future development of breast DWI methodologies.

## Conclusions

This meta-analysis shows that so far no conclusion can be drawn regarding which breast tumor tissue selection method outperforms in the differentiation of breast lesions by ADC values as calculated by the mono-exponential model of diffusion signal decay. Furthermore, there is no uniformity in the methodology of the included DWI breast studies. First, standardization and more accurate reporting of DWI protocols is needed to optimize the differentiation of breast lesions and to draw more definite conclusions in future research and finally in clinical care.

## Supporting information

S1 Checklist PRISMA 2009 checklist. (PDF)

S1 File Full search strategy. (PDF)
