# Supplementary material for: Diagnostic performance of breast tumor tissue selection in diffusion weighted imaging: A systematic review and meta-analysis
Source: PLoS One. 2020 May 6;15(5):e0232856. doi: 10.1371/journal.pone.0232856 (PMC7202642; doi:10.1371/journal.pone.0232856)
Supplement: S1 File — (PDF) [file pone.0232856.s002.pdf]

S2. Full search strategy.

1. PubMed: (“Breast Neoplasms” [Mesh] OR "Breast"[Mesh] OR mamma carcin*[tiab] OR mammary[tiab] OR mammo*[tiab] OR breast[tiab]) AND (“Diffusion Magnetic Resonance Imaging” [Mesh] OR "Diffusion Tensor Imaging"[Mesh] OR DWI[tiab] OR diffusion-weighted imag*[tiab] OR (("Diffusion"[Mesh] OR diffusion[tiab]) AND (“Magnetic Resonance Imaging” [Mesh] OR MRI[tiab] OR magnetic resonan*[tiab] OR MR[tiab] OR NMR[tiab])))

2. Embase: (('breast tumor'/exp OR 'breast'/exp OR (‘mamma carcin*’ OR mammary OR mammo* OR breast):ab,ti) AND ('diffusion weighted imaging'/exp OR 'diffusion tensor imaging'/exp OR (DWI OR ‘diffusion-weighted’):ab,ti OR (diffusion:ab,ti,de AND ('nuclear magnetic resonance imaging'/exp OR MRI:ab,ti OR ‘magnetic resonan*’:ab,ti OR MR:ab,ti OR NMR:ab,ti))) NOT 'conference abstract'/it)
